# Exogenous Ghrelin Could Not Ameliorate 3,4-methylenedioxymethamphetamine-induced Acute Liver Injury in The Rat: Involved Mechanisms

**DOI:** 10.22037/ijpr.2020.1100940

**Published:** 2020

**Authors:** Ravieh Golchoobian, Fatemeh Nabavizadeh, Mehrdad Roghani, Alireza Foroumadi, Maryam Izad, Maryam Bahrami, Hafseh Fanaei

**Affiliations:** a *Department of Physiology, School of Medicine, Tehran University of Medical Sciences, Tehran, Iran. *; b *Neurophysiology Research Center, Shahed University, Tehran, Iran. *; c *Department of Medicinal Chemistry, Faculty of Pharmacy, Tehran University of Medical Sciences, Tehran, Iran. *; d *Department of Immunology, School of Medicine, Tehran University of Medical Sciences Tehran, Iran. *; e *Department of Physiology, School of Medicine, Ahvaz Jundishapur University of Medical Sciences, Ahvaz, Iran.*

**Keywords:** Hepatotoxicity, 3, 4-methylenedioxymethamphetamine, Ghrelin, TNF-α, Apoptosis, Necrosis

## Abstract

MDMA (3,4-methylenedioxymethamphetamine, ecstasy) is often abused by youth as a recreational drug. MDMA abuse is a growing problem in different parts of the world. An important adverse consequence of the drug consumption is hepatotoxicity of different intensities. However, the underlying mechanism of this toxicity has not been completely understood. Ghrelin is a gut hormone with growth hormone stimulatory effect. It expresses in liver, albeit at a much lower level than in stomach, and exerts a hepatoprotective effect. In this study, we investigated hepatotoxicity effect of MDMA alone and its combination with ghrelin as a hepatoprotective agent. MDMA and MDMA+ ghrelin could transiently increase serum alanine aminotransferase (ALT) and aspartate aminotransferase (AST) followed by tissue necrosis. However, they could significantly decrease liver tumor necrosis factor-a (TNF-±) in both treatment groups. Unexpectedly, in MDMA treated rats, Bax, Bcl-xl, Bcl-2, Fas, Fas ligand (Fas-L), caspase 8, cytochrome c, caspase 3 gene expression, and DNA fragmentation were nearly unchanged. In addition, apoptosis in MDMA+ ghrelin group was significantly reduced when compared with MDMA treated animals. In all, MDMA could transiently increase serum transaminases and induce tissue necrosis and liver toxicity. Ghrelin, however, could not stop liver enzyme rise and MDMA hepatotoxicity. MDMA hepatotoxicity seems to be mediated via tissue necrosis than apoptotic and inflammatory pathways. Conceivably, ghrelin as an anti-inflammatory and anti-apoptotic agent may not protect hepatocytes against MDMA liver toxicity.

## Introduction

MDMA (3,4-methylenedioxymethamphetamine, ecstasy) is a synthetic drug derivative of amphetamine and is commonly known as love drug (1, 2). It is frequently used at ‘rave’ parties for vigorous dance without going into a state of fatigue (3). It is often abused by youth as a recreational drug (4). Nowadays, MDMA abuse in different parts of the world comes in epidemic proportions (5). The liver is a major target for MDMA-induced toxicity (4). MDMA hepatotoxicity is idiosyncratic in nature and can be manifested from mild spontaneously healed hepatitis (3, 6), to severe liver dysfunction (7, 8), or even a fulminant liver failure (3, 9-12). The mechanism of this liver injury is quite complex and rather unknown. There are various hypotheses in this regard including a possible direct effect of amphetamines and/or reactive metabolites, increased neurotransmitter efﬂux, biogenic amine oxidation, hyperthermia, mitochondrial impairment, and apoptosis (13). Among these potential mechanisms, apoptosis has been the center of attention in many researches (1, 14). Despite untoward sequels of MDMA, some researchers are still keen to use it for post-traumatic stress disorder and anxiety. MDMA has depicted to have immunomodulatory and neuroimmune characteristics (15) with contradictory responses in different settings (16-19).

Ghrelin is a peptide that was first purified from rat stomach and recognized as an endogenous ligand for the growth hormone secretagogue receptor. This orexigenic hormone is produced in endocrine cells of the stomach known as X/A-like cells in addition to other tissues like the liver (20, 21). Acute administration of MDMA increases endogenous serum ghrelin concentration with no significant change in serum growth hormone level (22). Ghrelin has also shown to have hepatoprotective (20, 23 and 24) and anti-inflammatory effects (21, 25 and 26). In sepsis, it decreases TNF-α level and can inhibit its hepatocellular dysfunction (27). Studies have also reported anti-apoptotic properties of ghrelin in muscle, intestine, and liver (24, 28-30). Therefore, based on anti-apoptotic, anti-inflammatory, and hepatoprotective effects of ghrelin we designed this study to investigate underlying MDMA hepatotoxical mechanisms and its combination with ghrelin on liver injury to see how this combination will be beneficial. 

## Experimental

*Materials*

MDMA (purity was 99.8%) was procured from the organic chemistry laboratory of Tehran medical sciences university pharmacy faculty (Tehran University of Medical Sciences, Tehran, Iran). Rat ghrelin was purchased from Sigma-Aldrich (St. Louis MO, USA), RiboEx total RNA was from GeneAll (Seoul, Korea), and cDNA synthesis kit and SYBER Premix Ex Taq™ were from Takara Bio Inc. (Otsu, Shiga, Japan), cell-death detection ELISA kit was from Roche (Mannheim, Germany), Rat TNF-α ELISA kit and protease inhibitor were from Sigma-Aldrich (St. Louis MO, USA). 

*Animals*

Forty-eight male adult Wistar rats weighing 200-250 g were obtained from animal house (Physiology Department, Tehran University of Medical Sciences, Tehran, Iran) and kept under standard conditions of temperature (22 °C ± 2) and light/dark cycle (12-hour) with free access to food and water. All experimental procedures were in accordance with the guidelines for the Care and Use of Laboratory Animals published by National Institutes of Health (NIH Publication No. 85-23, revised 1996) and approved by the research council of Tehran university of medical sciences.

*Study design*

The animals were randomly divided into four equal-sized groups of 1) MDMA (n = 12); the animals received single intraper-itoneal injection of MDMA (20 mg/kg, i.p.). 2) Control (n = 12); the animals received an equal intraperitoneal volume of normal saline as vehicle. 3) Ghrelin (n = 12); the animals received double apart doses of ghrelin (10 nmol/kg, i.p. with 3h gap). 4) MDMA+Ghrelin (n = 12); animals received MDMA (20 mg/kg, i.p.) followed by doubled apart ghrelin doses (10 nmol/kg, i.p. with 3h gap) 1 h post MDMA administration. The rats were then euthanized 6 h or 24 h following intervention to assess laboratory parameters. In all groups, half of the animals were anesthetized 6 h following intervention, and after blood sampling, the middle lobe of the liver was harvested to measure TNF-α. Eighteen hours after the initial blood collection, blood sampling was performed and tissue samples were taken out to apoptosis assay, quantitative real-time PCR, and histological evaluation for the other half of the animals. MDMA optimal dose adopted from our dose-response study accordingly (unpublished data). This dose has also been used in earlier similar studies (22, 31 and 32). Ghrelin dose was also obtained from our previous study (33). 

*Blood biochemistry studies*

Whole blood sample drew from the left animal cardiac ventricle and centrifuged at 3000 rpm for 10 min. Serum was then separated and stored at -70 °C. Serum alanine aminotransferase (ALT) and aspartate aminotransferase (AST) and alkaline phosphatase (ALP) were measured using Sapphire 800 auto analyzer (Cork, Ireland).

*Histological study*

Liver tissue excised under deep anesthesia and immersed in 10% buffered formalin. After paraffin embedding and sectioning of the tissue into 5 μm slices, all sections were stained with hematoxylin and eosin (H and E). Histological evaluation performed using light microscopy in a blinded manner. Liver cell necrosis was graded according to the following criteria: 0, absent; 1, mild; 2, moderate; and 3, severe (34, 35).

*Reverse transcription polymerase chain reaction (RT-PCR)*

Isolated liver tissues were snapped frozen in liquid nitrogen and stored at -70 °C until used. A hundred mg of liver fresh tissue was taken for RNA extraction using RiboEx Total RNA according to the manufacturer’s instructions. Total RNA concentration was then measured (NanoDrop 2000 Thermo Scientific, Wilmington, DE, USA). The samples were finally aliquoted and stored at -70 °C for further analysis. RNA extracts also treated with DNase I RNase-free solution to prevent any trace of genomic contamination. Afterwards, complementary DNA was synthesized from total RNA using cDNA synthesis kit according to the manufacturer’s instructions. Quantitative real-time PCR was performed by Step one plus Real-Time PCR System (Applied Biosystems, Carlsbad, CA, USA) based on the comparative threshold cycle method following manufacture’s protocol. GAPDH used as housekeeper genes to standardize gene expressions. The primer pairs were shown in Table 1. Relative gene expression of the treated group to control was calculated by 2^−ΔΔCt^ method. Primer design was performed using Primer 3 and BLAST (NCBI). 

*Preparation of tissue lysates*

Liver tissue was dissected out and homogenated in 10% concentration using a homogenizer (IKA, Germany) in cold RIPA lysis buffer containing 0.1% Triton X100, 0.1% sodium deoxycholate, 0.1% sodium dodecyl sulfate, and protease inhibitor cocktail (AEBSF, aprotinin, bestatin, E-64, leupeptin and EDTA), followed by centrifugation at 10000 ×*g* (5 min, 4 °C). Aliquoted supernatant was then stored at -70 °C.

*Assessment of tissue TNF-α level and apoptosis assay*

Quantitative TNF-α and cytoplasmic histone-associated DNA fragments of tissue lysates were determined according to kits manufacturer’s instructions. TNF-α and DNA fragmentation values were expressed as picogram per milliliter and optical density (OD) of each experimental group, respectively.

*Data analysis*

Data presented as mean ± SEM. Data normality was assessed with Kolmogorov-smirnov test. One-way ANOVA and Tukey post-hoc tests were used to compare means in different groups. Differences were considered statistically significant if *p* < 0.05. 

## Results

*Biochemical markers*

**Figure 1.**
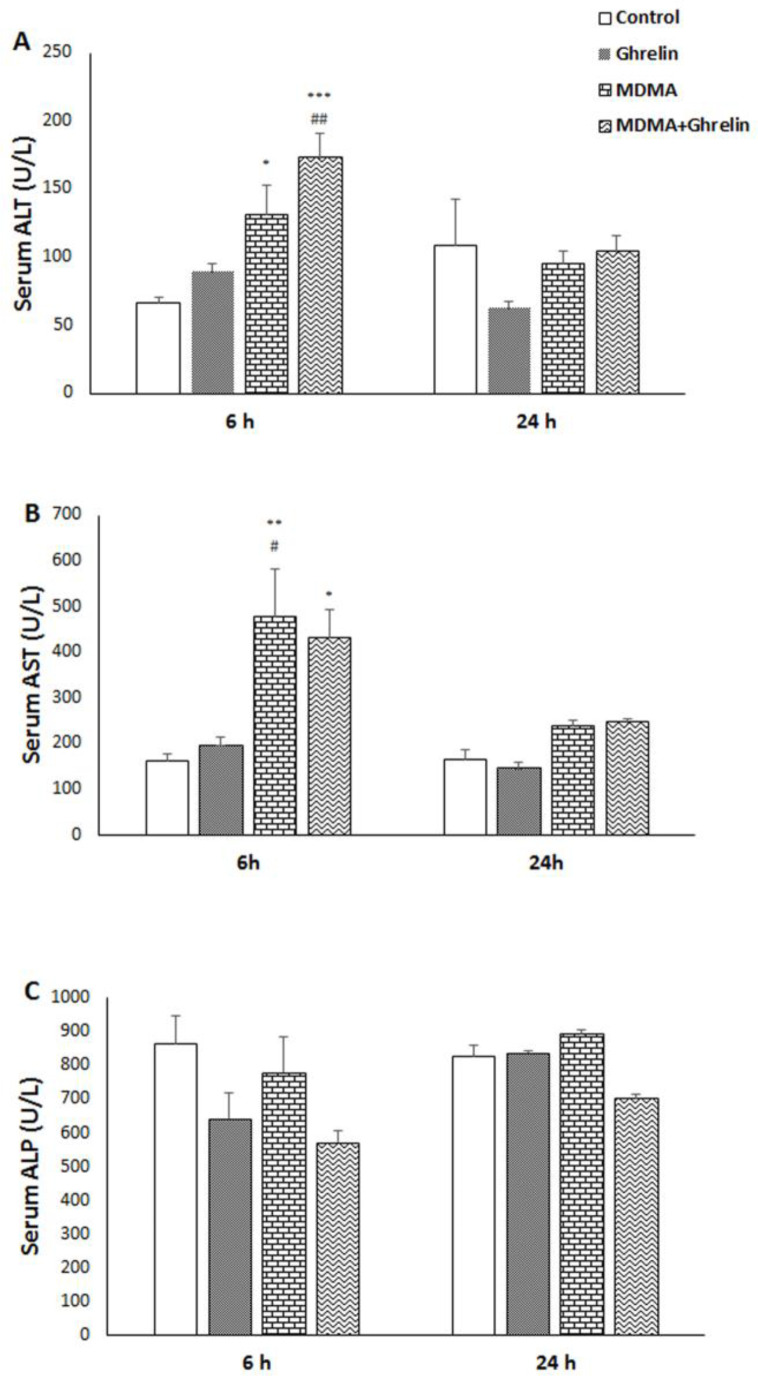
Serum (A) ALT, (B) AST and (C) ALP levels in control animals, ghrelin (10 nmol/kg*, *i.p. with 3 h apart) injected animals, MDMA (20 mg/kg, i.p.) injected animals and MDMA in combination with ghrelin. Levels of enzyme were measured 6 h and 24 h after intervention. Statistical comparison between groups was performed using analysis of variance followed by the Tukey test. ^*^p < 0.05, ^**^p < 0.01, ^***^p < 0.001 as compared to control group; ^#^*p* < 0.05, ^##^*p* < 0.01 as compared to ghrelin group

We first examined the effect of MDMA and MDMA in combination with ghrelin on rat liver function through the evaluation of serum levels of ALT, AST, and ALP by collecting blood 6 h and 24 h after MDMA administration. Serum ALT and AST levels of both MDMA (f (3, 20) = 10.01, *p* < 0.05, f (3, 20) = 6.84,* p *< 0.01, respectively) and MDMA+Ghrelin received animals significantly increased (f (3, 20) = 10.01, *p* < 0.001, f (3, 20) = 6.84, *p* < 0.05, respectively), with no significant changes in ALP level six hours after MDMA administration compared to the control group. Meanwhile, at 24 h post MDMA administration, ALT, AST, and ALP declined and nearly reached to control group in MDMA and MDMA+Ghrelin animals. No significant differences in ALT, AST, and ALP were noted in control and ghrelin groups at aforementioned time gap (Figure 1).

*Hepatic TNF-α measurement*

**Figure 2:**
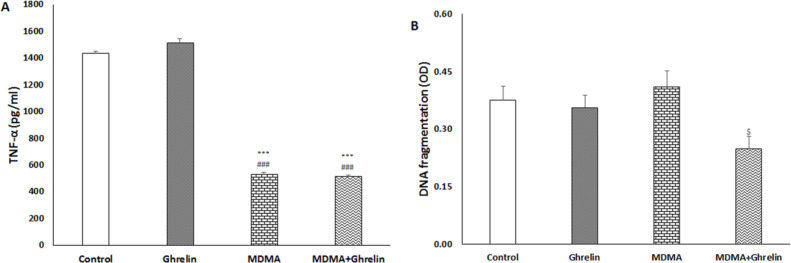
(A) TNF-α and (B) DNA fragmentation in control animals, ghrelin (10 nmol/kg, i.p. with 3 h apart) injected animals, MDMA (20 mg/kg, i.p.) injected animals and MDMA in combination with ghrelin at the same dosage exposure animals. Level of TNF-α was measured in liver tissue 6 h after intervention. DNA fragmentation was measured in liver tissue 24 h after intervention. Statistical comparison between groups was performed using analysis of variance followed by the Tukey test. ^***^p < 0.001 as compared to control group, ^###^*p* < 0.001 as compared to ghrelin group, ^$^*p* < 0.05 as compared to MDMA group

Liver tissue TNF-α of the rats treated with MDMA and MDMA+Ghrelin both significantly decreased (f (3, 17) = 434.21, *p* < 0.001) compared with the normal saline and ghrelin received animals. No significant differences in TNF-α level were noted between the control and ghrelin received rats, (Figure 2A).

*Hepatic DNA fragmentation*

Liver tissue apoptosis was determined with a cytoplasmic histone-associated DNA fragments assay as a valid apoptosis indicator (36) at 24 h post MDMA administration. In the MDMA+Ghrelin received rats apoptosis significantly was reduced compared with the MDMA received animals (f (3, 17) = 3.06, *p* < 0.05). Cytoplasmic histone-associated DNA fragments in MDMA and ghrelin received animals did not show significant difference when compared with the control group (Figure 2B).

*Liver apoptosis-related genes expression*

**Figure 3:**
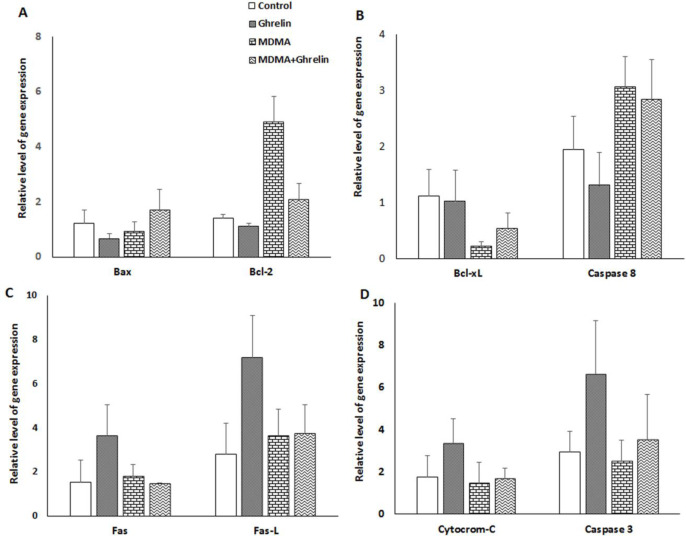
The relative gene expression level of (A) Bax and Bcl-2, (B) Bcl-xL and caspase 8, (C) Fas and Fas-L, (D) cytochrome c and caspase 3 in control animals, ghrelin (10 nmol/kg*, *i.p. with 3 h apart) exposure animals, MDMA (20 mg/kg, i.p.) exposure animals and MDMA in combination with ghrelin. The relative expression level of each gene was determined by real-time polymerase chain reaction. Statistical comparison between groups was performed using analysis of variance followed by the Tukey test. There were no statistical differences between groups

The expression level of 8 genes associated with apoptosis which we selected did not significantly differ in various groups, but the expression of anti-apoptotic protein, Bcl-2, in MDMA and MDMA+Ghrelin, was 3.5-fold and 1.5-fold higher, but it was non-significant versus control, respectively (Figure 3A). mRNA level of Bcl-xl as a survival protein was lower (non-significant) in the animals treated with MDMA alone and in combination with ghrelin (0.2-fold and 0.5-fold, respectively) (Figure 3B). There were no significant differences in the levels of Fas, Fas-L cytochrome-c, caspase 8 and caspase 3 gene expression among the groups (Figures 3C and 3D). 

*Histological examination*

Liver tissues in the control group showed normal architecture. However, in both MDMA and MDMA+ ghrelin received animals histological- induced changes were noted compared with the control group. In addition, hepatocytes necrosis were significantly higher in the MDMA and MDMA+ ghrelin received animals (*p* < 0.05, f (3, 12) = 6.223) than the control. The ghrelin received animals showed no morphological change.

## Discussion

To the best of our knowledge, this is the first study that investigated the possible mechanisms of MDMA hepatotoxicity and protective effects of MDMA+ghrelin administration on the rat liver tissues. In our study, MDMA could transiently increase liver enzymes, an indicative of MDMA-induced hepatotoxicity. MDMA could also remarkably reduce hepatic TNF-α level with less influence on programed cell death and apoptosis-related gene expression despite the focal hepatic necrosis. Ghrelin administration post MDMA challenge neither suppress MDMA-induced hepatic necrosis and liver enzyme rise nor alleviate MDMA immunosuppressive effects, in spite of significant DNA fragmentation and unchanged apoptosis- related genes expressions.

Significant transient increases in serum ALT and AST levels in MDMA and MDMA in combination with the ghrelin groups as compared to the control group were observed. Additionally, ghrelin showed more elevation of serum ALT and less elevation of serum AST versus those elevations induced by MDMA. Increased AST and ALT activities as indices of MDMA-induced cytotoxicity in rat hepatocytes have been previously demonstrated (37). It has also been reported that MDMA-induced hepatotoxicity is associated with hepatic transaminases elevation in humans and laboratory animals (3, 6 and 38). Transaminases rises are indicative of necrosis, since ALT and AST are two of the most reliable indicators of hepatic injury and necrosis (39). Our results showed that co-administration of MDMA and ghrelin increases ALT level, as a specific marker for hepatic injury, and decreased AST levels as compared to the control. In fact, ghrelin could not prevent MDMA-induced hepatic injury. Since AST is also present in other tissues (39), ghrelin-induced reduction in AST may be indicative of the beneficial effect of ghrelin on the other tissues. It should be noted that when baseline values were compared between treated and control groups, ghrelin was not able to completely normalize MDMA-induced AST elevation. 

In the present study, MDMA could reduce liver TNF-α level when compared to the control group. Ghrelin did not alter MDMA-induced TNF-α reduction. MDMA inhibitory effect on TNF-α level has been reported in the presence of pre and post immune challenge, but surprisingly, in this study for the first time we saw that single dose MDMA could suppress TNF-α production in the absence of any immune challenge. Interestingly, several contradictory studies are found in the literature. For instance, Cerretani *et al.* reported a strong positive expression for TNF-α production post MDMA use (31). However, Connor et al observed no change in TNF-α levels following MDMA administration within 2 h to 24 h (16-19, 40). One possible mechanism refers to vagus nerve and peripheral nicotinic acetylcholine receptors that mediate MDMA- induced TNF-α inhibition (17). Reportedly, ghrelin has a wide array of anti-inflammatory activities in endotoxin shock and sepsis models (41, 42). In our study, MDMA seems to have strong inhibitory effects on TNF-α production that cannot be further potentiated by ghrelin administration (Figure 2A).

The pro-inflammatory cytokine TNF-α is regarded as an important signaling molecule in initiating and coordinating a range of immune-related responses against pathogenic agents (18). Therefore, MDMA-induced TNF-α reduction may affect host resistance to infection (43-47). Additionally, it has been suggested that immunosuppressive property of MDMA is at least partly responsible for MDMA-induced hepatotoxicity (4). Since, liver is an important organ involved in detoxification and likely to be injured by ingested toxins or drugs, it is highly capable of regeneration for its survival (48). Several studies have depicted TNF-α role in tissue regeneration (49, 50). Thus, in our study reduced TNF-α level may indicate MDMA-induced regeneration defect following toxic injury. Anti-TNF-α agents have shown to be toxic for liver tissues with unknown underlying mechanisms (51).

Despite *in-vitro* MDMA pro-apoptotic effects on rat hepatocytes, in our *in-vivo* study, we could not show MDMA pro-apoptotic activities. This finding may be due to different MDMA dose, way of administration, duration of exposure and *in-vivo* *vs.* *in-vitro* disparities on apoptosis induction (1, 19 and 31). Apoptotic effect of MDMA may occur in *in-vivo* setting if MDMA is used for repeated doses or for chronic purposes. In support of this claim, Warren *et al.* has shown that apoptosis of the liver occurred just 72 h after MDMA administration (52).

In the present study, real- time RT-PCR approach was used to assess molecular mechanisms that may be involved in the apoptotic process. Gene expressions varied between the groups; however, it failed to reach a significant level. It has been reported that an up-regulation of bcl-2 gene can prevent methamphetamine-induced apoptosis of immortalized neuron cells (53). MDMA-treated animals demonstrated a 3.5-fold increase of Bcl-2 and a 0.8-fold decrease of Bax expression, probably indicating a defensive mechanism of the liver cells to resists MDMA-induced programed cell death. As far as we know, no study has examined the effects of MDMA on Fas/Fas-L expression in liver. The Fas ⁄Fas-L system is an important pathway for programed cell death in the hepatocytes (54). Another interesting finding of this study was that the gene expression of Fas/Fas-L in the liver remained unchanged following MDMA expousure. Despite no significant results for MDMA-induced apoptosis, we observed a significant reduction of apoptosis in MDMA+Ghrelin as compared to MDMA group. These effects could be related in part to the fact that expression of anti-apoptotic cell regulator Bcl-xl decreased (non-significant) by 20% in the MDMA and restored (non-significant) to 50% of the control with MDMA+Ghrelin treatment. It has been demonstrated that ghrelin treatment following irradiation increase the Bcl-xl protein expression levels which was followed by apoptosis reduction (30). Ghrelin has shown to prevent apoptosis of cardiomyocytes and endothelial cells through stimulating anti-apoptotic intracellular signalling pathways, such as activation of extracellular-signal-regulated kinase-1 and -2, protein kinase B (Akt), and tyrosine phosphorylation of intracellular proteins (29). This may indicate that ghrelin via post-translational modifications exerts anti-apoptotic effect in liver. Previously, it has been hypothesized that apoptosis plays a major role in MDMA-induced hepatotoxicity; however, our study shows that ghrelin, being an anti-apoptotic agent fails to diminish MDMA-induced hepatic injury.

**Figure 4.**
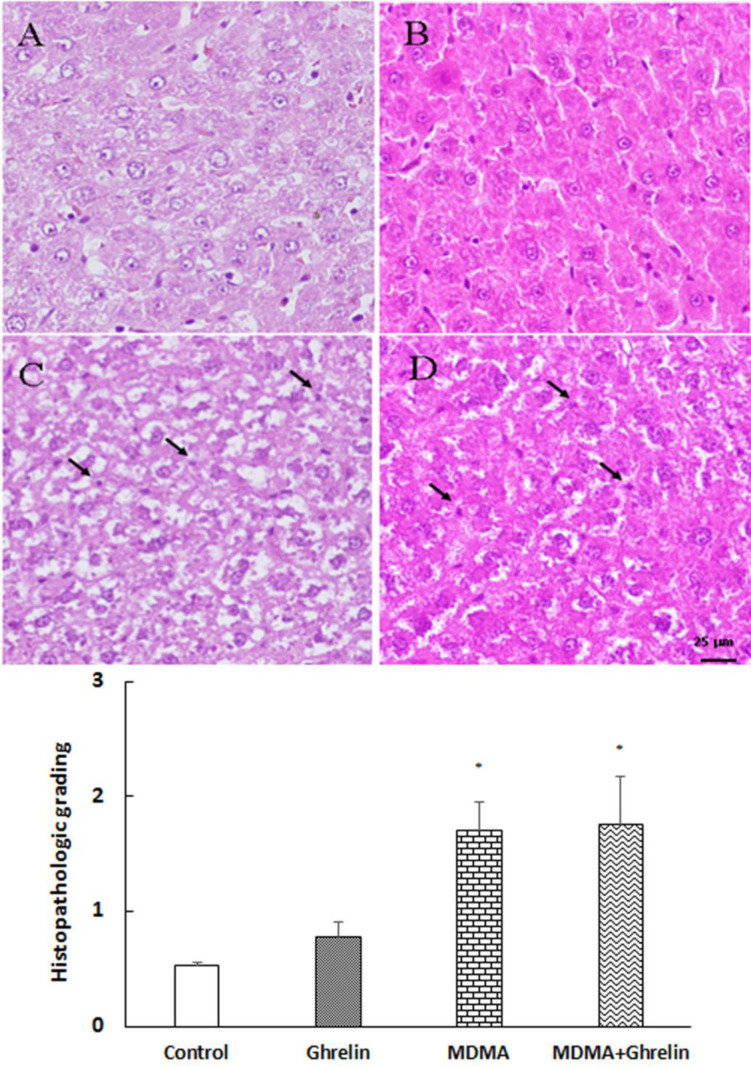
Histological sections of the rat liver from (A) control, (B) ghrelin (10 nmol/kg, i.p. with 3 h apart) injected animals, (C) MDMA (20 mg/kg, i.p.) injected animals and (D) MDMA in combination with ghrelin at the same dosage exposure animals were stained with Hematoxylin and Eosin stain. Magnification 400x. The histological changes were scored according to the following criteria: 0, absent; 1, mild; 2, moderate; and 3, severe hepatocellular necrosis. Statistical comparison between groups was performed using analysis of variance followed by the *Tukey* test. ^*^p < 0.05 as compared to control group

Tweny four hours follwing MDMA administration, histological analysis showed a significant increase in necrosis of hepatocytes and ghrelin administration did not improve the tissue histology (Figure 4). Several clinical studies have suggected a more prominent role of necrosis than apoptosis in causing MDMA-induced hepatotoxity (55). Additionaly, previous *in-vitro* studies have demonstreted that MDMA triggers necrosis rather than apoptosis by increasing the temperature of the medium (55, 56). The rise in temperature has been shown to induce oxidative stress and deplete cellular ATP which in turn may lead to hepatic necrosis (55). Since, necrosis and apoptosis are key features of liver injury (57), it is assumed that MDMA-induced necrosis may be attributed to transient increases in liver enzymes. However, further studies are warranted to clarify the role of necrosis in MDMA-induced liver injury. In the present study ghrelin did not show any hepatoprotective effect in MDMA-induced hepatotoxicity since, ghrelin administration did not decrease nighter liver enzymes and necrosis elevation nor TNF-α reduction.

**Table 1:** Oligonucleotide primers used in qRT-PCR

***mRNA***	***Forward (5’-3’)***	***Reverse (5’-3’)***
*Bax*	CTCAAGGCCCTGTGCACTAAA	GGGGGTCCCGAAGTAGGAA
*Bcl-2*	CATCGCTCTGTGGATGACTGA	CTGGGGCCATATAGTTCCACAA
*Bcl-xl*	GCAGTCAGCCAGAACCCTATC	GGGCTCAACCAGTCCATTGT
*Cytocrom c*	CTTGGGCTAGAGAGCGGGA	GTGGCACTGGGCACACTTTT
*Caspase 3*	GAGCTTGGAACGCGAAGAAA	GAGTCCATCGACTTGCTTCCA
*Caspase 8*	AAAGCCCAGGTTTCTGCCTA	ATCAAGCAGGCTCGAGTTGTC
*Fas*	AGGGCATGGTTTAGAAGTGG	GTGCAAGGCTCAAGGATGTC
*Fas-L*	ACTCCGTGAGTTCACCAACC	TAAGTGGGCCACACTCCTTG
*GAPDH*	TCTCTGCTCCTCCCTGTTCTA	GGTAACCAGGCGTCCGATAC

## Conclusion

In our study, MDMA caused transient increase in serum transaminase levels that was associated with reduction of TNF-α and increased necrosis of hepatic tissue. Moreover, MDMA made no obvious perturbation in the mitochondrial-induced apoptosis pathway. Additionally, administration of exogenous ghrelin could not ameliorate MDMA-induced acute liver injury.

## References

[1] Montiel-Duarte C, Varela-Rey M, Osés-Prieto JA, López-Zabalza MaJ, Beitia G, Cenarruzabeitia E, Iraburu MaJ (2002). 3, 4-Methylenedioxymethamphetamine (“Ecstasy”) induces apoptosis of cultured rat liver cells. Biochim. Biophys. Acta.

[2] Hall A, Henry J (2006). Acute toxic effects of ‘Ecstasy’(MDMA) and related compounds: overview of pathophysiology and clinical management. Br. J. Anaesth..

[3] Ellis A, Wendon J, Portmann B, Williams R (1996). Acute liver damage and ecstasy ingestion. Gut.

[4] Antolino-Lobo I, Meulenbelt J, van den Berg M, van Duursen MB (2011). A mechanistic insight into 3, 4-methylenedioxymethamphetamine (“ecstasy”)-mediated hepatotoxicity. Vet. Q..

[5] Karch SB (2011). A historical review of MDMA. Open Forensic Sci. J..

[6] Dykhuizen R, Brunt P, Atkinson P, Simpson J, Smith C (1995). Ecstasy induced hepatitis mimicking viral hepatitis. Gut.

[7] Henry J, Jeffreys K, Dawling S (1992). Toxicity and deaths from 3, 4-methylenedioxymethamphetamine (“ecstasy”). Lancet.

[8] Milroy C, Clark J, Forrest A (1996). Pathology of deaths associated with” ecstasy” and” eve” misuse. J. Clin. Pathol..

[9] Brauer LH, De Wit H (1997). High dose pimozide does not block amphetamine-induced euphoria in normal volunteers. Pharmacol. Biochem. Behav..

[10] Caballero F, Lopez-Navidad A, Cotorruelo J, Txoperena G (2002). Ecstasy-induced brain death and acute hepatocellular failure: multiorgan donor and liver transplantation. Transplantation.

[11] Garbino J, Henry J, Mentha G, Romand JA (2001). Ecstasy ingestion and fulminant hepatic failure: liver transplantation to be considered as a last therapeutic option. Vet. Hum. Toxicol..

[12] Liechti ME, Kunz I, Kupferschmidt H (2005). Acute medical problems due to Ecstasy use Case-series of emergency department visits. Swiss Med. Wkly..

[13] Carvalho M, Carmo H, Costa VM, Capela JP, Pontes H, Remião F, Carvalho F, de Lourdes Bastos M (2012). Toxicity of amphetamines: an update. Arch. Toxicol..

[14] Montiel-Duarte C, Ansorena E, López-Zabalza MJ, Cenarruzabeitia E, Iraburu MJ (2004). Role of reactive oxygen species, glutathione and NF-κB in apoptosis induced by 3, 4-methylenedioxymethamphetamine (“Ecstasy”) on hepatic stellate cells. Biochem. Pharmacol..

[15] Stankevicius D, Ferraz-de-Paula V, Ribeiro A, Pinheiro ML, de Oliveira APL, Damazo AS, Lapachinske SF, Moreau RL, de Lima WT, Palermo-Neto J (2012). 3, 4-methylenedioxymethamphetamine (ecstasy) decreases inflammation and airway reactivity in a murine model of asthma. Neuroimmunomodulation.

[16] Connor TJ, Dennedy MC, Harkin A, Kelly JP (2001). Methylenedioxymethamphetamine-induced suppression of interleukin-1β and tumour necrosis factor-α is not mediated by serotonin. Eur. J. Pharmacol..

[17] Camarasa J, Ros C, Pubill D, Escubedo E (2010). Tumour necrosis factor alpha suppression by MDMA is mediated by peripheral heteromeric nicotinic receptors. Immunopharmacol. Immunotoxicol..

[18] Connor TJ, Harkin A, Kelly JP (2005). Methylenedioxymethamphetamine suppresses production of the proinflammatory cytokine tumor necrosis factor-α independent of a β-adrenoceptor-mediated increase in interleukin-10. J. Pharmacol. Exp. Ther..

[19] Connor TJ, Kelly JP, McGee M, Leonard BE (2000). Methylenedioxymethamphetamine (MDMA; Ecstasy) suppresses IL-1β and TNF-α secretion following an in-vivo lipopolysaccharide challenge. Life Sci..

[20] Çetin E, Kanbur M, Çetin N, Eraslan G, Atasever A (2011). Hepatoprotective effect of ghrelin on carbon tetrachloride-induced acute liver injury in rats. Regul. Pept..

[21] Delporte C (2013). Structure and physiological actions of ghrelin. Scientifica.

[22] Kobeissy FH, Jeung JA, Warren MW, Geier JE, Gold MS (2008). Preclinical Study: Changes in leptin, ghrelin, growth hormone and Neuropeptide‐Y after an acute model of MDMA and methamphetamine exposure in rats. Addict. Biol..

[23] Li Y, Hai J, Li L, Chen X, Peng H, Cao M, Zhang Q (2013). Administration of ghrelin improves inflammation, oxidative stress, and apoptosis during and after non-alcoholic fatty liver disease development. Endocrine.

[24] Moreno M, Chaves JF, Sancho-Bru P, Ramalho F, Ramalho LN, Mansego ML, Ivorra C, Dominguez M, Conde L, Millán C (2010). Ghrelin attenuates hepatocellular injury and liver fibrogenesis in rodents and influences fibrosis progression in humans. Hepatology.

[25] Li WG, Gavrila D, Liu X, Wang L, Gunnlaugsson S, Stoll LL, McCormick ML, Sigmund CD, Tang C, Weintraub NL (2004). Ghrelin inhibits proinflammatory responses and nuclear factor-κB activation in human endothelial cells. Circulation.

[26] Dembinski A, Warzecha Z, Ceranowicz P, Konturek P, Tomaszewska R, Stachura J, Konturek S (2003). Ghrelin attenuates the development of acute pancreatitis in rat. J. Physiol. Pharmacol..

[27] Jacob A, Rajan D, Pathickal B, Balouch I, Hartman A, Wu R, Zhou M, Wang P (2010). The inhibitory effect of ghrelin on sepsis-induced inflammation is mediated by the MAPK phosphatase-1. Int. J. Mol. Med..

[28] Yu A, Pei X, Sin T, Yip S, Yung B, Chan L, Wong C, Siu P (2014). Acylated and unacylated ghrelin inhibit doxorubicin‐induced apoptosis in skeletal muscle. Acta Physiol..

[29] Baldanzi G, Filigheddu N, Cutrupi S, Catapano F, Bonissoni S, Fubini A, Malan D, Baj G, Granata R, Broglio F (2002). Ghrelin and des-acyl ghrelin inhibit cell death in cardiomyocytes and endothelial cells through ERK1/2 and PI 3-kinase/AKT. J. Cell Biol..

[30] Wang Z, Yang WL, Jacob A, Aziz M, Wang P (2015). Human ghrelin mitigates intestinal injury and mortality after whole body irradiation in rats. PLoS One.

[31] Cerretani D, Bello S, Cantatore S, Fiaschi A, Montefrancesco G, Neri M, Pomara C, Riezzo I, Fiore C, Bonsignore A (2011). Acute administration of 3, 4-methylenedioxymethamphetamine (MDMA) induces oxidative stress, lipoperoxidation and TNFα-mediated apoptosis in rat liver. Pharmacol. Res..

[32] Aguirre N, Barrionuevo M, Ramírez MJ, Del Río J, Lasheras B (1999). α-Lipoic acid prevents 3, 4-methylenedioxy-methamphetamine (MDMA)-induced neurotoxicity. Neuroreport.

[33] Jahromi MG, Nabavizadeh F, Vahedian J, Nahrevanian H, Dehpour AR, Zare-Mehrjardi A (2010). Protective effect of ghrelin on acetaminophen-induced liver injury in rat. Peptides.

[34] Camargo CA, Madden JF, Gao W, Selvan RS, Clavien P (1997). Interleukin-6 protects liver against warm ischemia/reperfusion injury and promotes hepatocyte proliferation in the rodent. Hepatology.

[35] Lin X, Liu X, Huang Q, Zhang S, Zheng L, Wei L, He M, Jiao Y, Huang J, Fu S (2012). Hepatoprotective effects of the polysaccharide isolated from Tarphochlamys affinis (Acanthaceae) against CCl 4-induced hepatic injury. Biol. Pharm. Bull..

[36] Yu Z, Zhang L, Wu D, Liu F (2005). Anti-apoptotic action of zearalenone in MCF-7 cells. Ecotoxicol. Environ. Safe..

[37] Beitia G, Cobreros A, Sainz L, Cenarruzabeitia E (1999). 3, 4-Methylenedioxymethamphetamine (ecstasy)-induced hepatotoxicity: effect on cytosolic calcium signals in isolated hepatocytes. Liver.

[38] Beitia G, Cobreros A, Sainz L, Cenarruzabeitia E (2000). Ecstasy-induced toxicity in rat liver. Liver.

[39] Giboney PT (2005). Mildly elevated liver transaminase levels in the asymptomatic patient. Am. Fam. Physician..

[40] Connor TJ, Kelly JP, Leonard BE (2000). An assessment of the acute effects of the serotonin releasers methylenedioxymethamphetamine, methylenedioxyamphetamine and fenfluramine on immunity in rats. Immunopharmacology.

[41] Dixit VD, Schaffer EM, Pyle RS, Collins GD, Sakthivel SK, Palaniappan R, Lillard JW, Taub DD (2004). Ghrelin inhibits leptin-and activation-induced proinflammatory cytokine expression by human monocytes and T cells. J. Clin. Invest..

[42] Wu R, Dong W, Cui X, Zhou M, Simms HH, Ravikumar TS, Wang P (2007). Ghrelin down-regulates proinflammatory cytokines in sepsis through activation of the vagus nerve. Ann. Surg..

[43] Pasparakis M, Alexopoulou L, Episkopou V, Kollias G (1996). Immune and inflammatory responses in TNF alpha-deficient mice: a critical requirement for TNF alpha in the formation of primary B cell follicles, follicular dendritic cell networks and germinal centers, and in the maturation of the humoral immune response. J. Exp. Med..

[44] Keane J (2005). TNF-blocking agents and tuberculosis: new drugs illuminate an old topic. Rheumatology.

[45] Strangfeld A, Listing J, Herzer P, Liebhaber A, Rockwitz K, Richter C, Zink A (2009). Risk of herpes zoster in patients with rheumatoid arthritis treated with anti–TNF-α agents. JAMA.

[46] Furst DE (2010). The risk of infections with biologic therapies for rheumatoid arthritis. Semin. Arthritis Rheum..

[47] Boyle NT, Connor TJ (2010). Methylenedioxymethamphetamine (‘Ecstasy’)-induced immunosuppression: a cause for concern? Br. J. Pharmacol..

[48] Taub R (2004). Liver regeneration: from myth to mechanism. Nat. Rev. Mol. Cell Biol..

[49] Bradham CA, Plümpe J, Manns MP, Brenner DA, Trautwein C I (1998). TNF-induced liver injury. Am. J. Physiol. Gastrointest. Liver Physiol..

[50] Schwabe RF, Brenner DA (2006). Mechanisms of liver injury TNF-α-induced liver injury: role of IKK, JNK, and ROS pathways. Am. J. Physiol. Gastrointest. Liver Physiol.

[51] French JB, Bonacini M, Ghabril M, Foureau D, Bonkovsky HL (2016). Hepatotoxicity associated with the use of anti-TNF-α agents. Drug Saf..

[52] Warren MW, Kobeissy FH, Liu MC, Svetlov SI, Hayes RL, Gold MS, Wang KK (2006). Ecstasy toxicity: a comparison to methamphetamine and traumatic brain injury. J. Addict. Dis..

[53] Cadet JL, Ordonez SV, Ordonez JV (1997). Methamphetamine induces apoptosis in immortalized neural cells: Protection by the proto-oncogene, bcl-2. Synapse.

[54] Bortolami M, Kotsafti A, Cardin R, Farinati F (2008). Fas/Fas-L system, IL-1β expression and apoptosis in chronic HBV and HCV liver disease. J. Viral Hepat..

[55] Da Silva DD, Carmo H, Lynch A, Silva E (2013). An insight into the hepatocellular death induced by amphetamines, individually and in combination: the involvement of necrosis and apoptosis. Arch. Toxicol..

[56] Pontes H, Sousa C, Silva R, Fernandes E, Carmo H, Remião F, Carvalho F, Bastos ML (2008). Synergistic toxicity of ethanol and MDMA towards primary cultured rat hepatocytes. Toxicology.

[57] Schulze-Bergkamen H, Schuchmann M, Fleischer B, Galle PR (2006). The role of apoptosis versus oncotic necrosis in liver injury: facts or faith? J. Hepatol..

